# Peritoneal mesothelioma with invasion of the urinary bladder 20 years post radiation therapy for colon adenocarcinoma

**DOI:** 10.1093/jscr/rjab392

**Published:** 2021-09-15

**Authors:** Nicole Ong Mingjing, Abdul Samad Asamu, Keith Pace, Simon Bugeja

**Affiliations:** Department of Surgery, Mater Dei Hospital, Msida, Malta; Department of Surgery, Mater Dei Hospital, Msida, Malta; Department of Urology, Mater Dei Hospital, Msida, Malta; Department of Urology, Mater Dei Hospital, Msida, Malta

## Abstract

We present the case of a 66-year-old male with a history of iatrogenic bladder injury and radiation therapy for colon adenocarcinoma 20 years prior. A computed tomography of the thorax, abdomen and pelvis, reported a presacral mass with invasion to the bladder, ureters and lymph nodes. An initial bladder biopsy was histologically inconclusive. A later biopsy taken during emergency bypass surgery for small bowel obstruction, while the patient was positive for coronavirus disease of 2019, concluded that the mass was a peritoneal mesothelioma of epithelioid origin. The combination management of cytoreductive surgery with intraperitoneal chemotherapy has been a near universal standard of care for epithelioid peritoneal mesothelioma provided that the patient is an appropriate candidate for surgical interventions. To the best of our knowledge, this is the first case report of malignant peritoneal mesothelioma as a second primary carcinoma, many years after exposure to radiation therapy for colon adenocarcinoma.

## INTRODUCTION

Peritoneal mesothelioma is a rare and fatal malignancy accounting for 30% of all mesotheliomas, with a prevalence of 0.3–0.4 and 0.9–1.7 per 100 000 population among females and males [1]. It has no fully known clinical characteristic for early detection and is often diagnosed at an advanced stage [2].

Although asbestos exposure remains the most significant risk factor, radiation exposure has been recently focused on. Advances in oncological managements have contributed to an increased life expectancy among cancer survivors leading to an increased in second primary malignancy incidences. Cases of mesothelioma as second primaries arising 10 years after radiotherapy have been reported in cervical and testicular cancers, but not after colon adenocarcinoma [3, 4].

## CASE REPORT

A 66-year-old-heavy male smoker, without asbestos exposure, had multiple hospital admissions for urological management after an iatrogenic bladder injury during an abdominal perineal resection for colon adenocarcinoma performed 20 years ago. He received radiotherapy after surgery and remained in remission.

Secondary to radiotherapy, he had a reduced bladder volume, with resultant vesicoureteric reflux, bilateral hydronephrosis and episodes of acute on chronic kidney injuries requiring bilateral permanent nephrostomies insertion.

**Figure 1:**
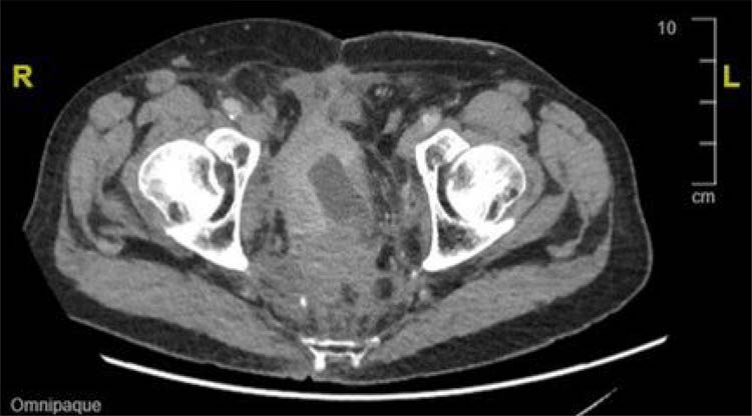
CT image showing an extensive mass in the presacral region with invasion to the urinary bladder and both ureters.

During an admission for deep vein thrombosis post lithotripsy of urethral calculi, a computed tomography (CT) of the thorax, abdomen and pelvis showed an extensive mass in the presacral region with invasion of the urinary bladder and orifices of both ureters with enlarged lower paraaortic and pelvic lymph nodes (Fig. 1). A urinary bladder biopsy obtained during a rigid cystoscopy showed an indeterminate invasive carcinoma.

A repeat rigid cystoscopy to obtain further biopsies was planned. Unfortunately, in the interim, the patient presented again with urosepsis, related to his bilateral nephrostomies, for which he was treated with high dose meropenem and teicoplanin for *Pseudomonas aeruginosa*. During this admission, he also tested positive for severe acute respiratory syndrome coronavirus 2 (SARS-CoV-2) on a reverse transcriptase-polymerase chain reaction test.

While being treated for urosepsis in the coronavirus disease (COVID) ward, he complained of a 3-day history of inability to open bowels, decreased appetite and abdominal pain. He was lethargic, his abdomen distended and a digital stoma examination was unremarkable. Laxatives were administered to no effect.

**Figure 2:**
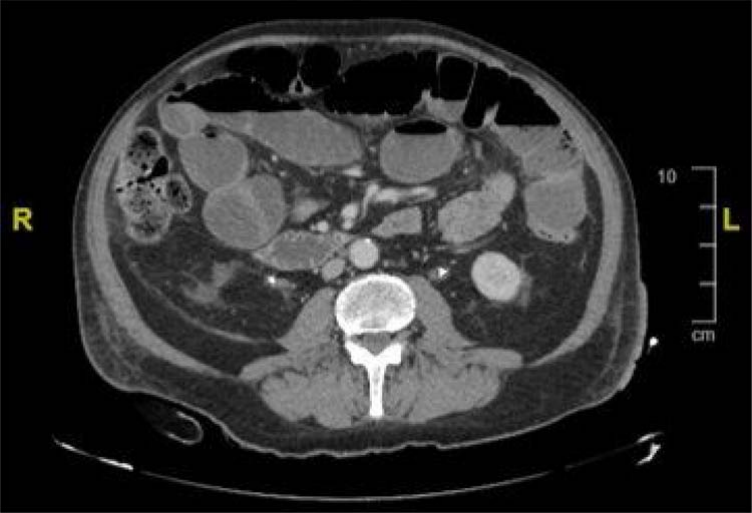
CT image showing distended bowel loops indicating bowel obstruction caused by the pelvic mass.

CT and magnetic resonance imaging (MRI) of the abdomen and pelvis indicated that a loop of small bowel was caught up in the pelvic mass resulting in a mechanical small bowel obstruction (Fig. 2).

An ileo-ascending colon bypass and change of nephrostomies were performed and fashioned in a COVID-dedicated operating theatre. During the procedure, the small bowel was found to be distended up to the ileum, without any evidence of ischemia or perforation. The distended ileum had entered the pelvic mass while the terminal ileum distal to the mass was collapsed. An ileul loop was anastomosed to the ascending colon and a biopsy from the pelvic mass was taken. Unfortunately, no images were taken during the procedure due to restrictions and limitations in the COVID theatre.

The biopsy showed discohesive infiltrate of plasmacytoid neoplastic cells with eccentrically-placed, bland nuclei and abundant eosinophilic cytoplasm with occasional binucleate cells present.

Immunohistochemistry showed strong and diffuse expression of WT1 and D2-40 together with more focal expression of calretinin, confirming a mesothelial phenotype. Strong and diffuse expression of BRCK, CK7 and vimentin were also seen with occasional cells express CD34 while Ki67 index was low. These findings were in-keeping with epithelioid mesothelioma without trace of sarcomatoid differentiation.

An opinion was sought from a tertiary referral center in the UK for the possibility of extirpative surgery. However, the disease was surgically unresectable with curative intent and palliative chemotherapy was therefore offered. Due to the possible adverse effects and limited potential benefit, he opted for symptomatic and conservative management instead.

Three months after the initial presentation, he remains with bilateral nephrostomies and had recently undergone another small bowel bypass for another episode of intestinal obstruction.

## DISCUSSION

Malignant peritoneal mesothelioma (MPM) is the second most common site for mesothelioma. However, it is an understudied disease as the majority of clinical studies focus primarily on the pulmonary variant. Mesothelioma commonly affects males aged over 65 years, smokers and those exposed to asbestos and radiation.

According to a 2014 study, Malta had one of the highest mesothelioma rates behind the UK, Australia, the Netherlands and New Zealand at 2.08 per 100 000 men [5]. Since the introduction of the 2005 European Union directive mandating the ban of new chrysotile use, a decreased rate from higher than 3 per 100 000 in 2005–2006 to 1.06 in 2012, was observed [1].

MPM as a second cancer >10 years after radiotherapy was reported in a cohort study performed by Farioli *et al.* in 2016 [6]. Rare cases of synchronous MPM with colonic adenocarcinoma and MPM after radiation therapy for reproductive malignancies have also been previously reported [3, 4, 7].

MPM has a poor prognosis of <5–12 months survival without treatment [8]. Chemotherapy alone has potential palliative benefit but very little impact on survival [9]. One of the first published management strategies described a combination regimen using cytoreductive surgery (CRS) and doxorubicin-based systemic chemotherapy, which provided a median survival of 22 months [10]. CRS together with hyperthermic intraperitoneal chemotherapy (HIPEC) later became the standard of care. A meta-analysis published in 2011 concluded that this combination is effective in operable MPM with an improved 53 months median survival and a 47% 5-year overall survival rate [11].

A review article published in 2018 reported greater benefit from CRS, HIPEC and normothermic intraperitoneal chemotherapy long-term (NIPEC-LT) in combination, with a 5-year survival rate of 70% [12]. Due to logistical limitations and the morbidity of surgery, the majority of MPM patients worldwide are offered palliative care or systemic chemotherapy. Careful patient selection is also critical for combination treatment. MPM patients in good general health, with epithelial histologic type, and without concerning CT features have a more favorable outcome [12].

Hydroureter, bowel obstruction and large tumor size were concerning radiologic features seen in our patient. Hence, chemotherapy and palliative care were offered in line with the Peritoneal Surface Oncology Group International recommendations [2].
